# Artificial intelligence‐assisted automatic and index‐based microbial single‐cell sorting system for One‐Cell‐One‐Tube

**DOI:** 10.1002/mlf2.12047

**Published:** 2022-12-18

**Authors:** Zhidian Diao, Lingyan Kan, Yilong Zhao, Huaibo Yang, Jingyun Song, Chen Wang, Yang Liu, Fengli Zhang, Teng Xu, Rongze Chen, Yuetong Ji, Xixian Wang, Xiaoyan Jing, Jian Xu, Yuandong Li, Bo Ma

**Affiliations:** ^1^ CAS Key Laboratory of Biofuels, Shandong Key Laboratory of Energy Genetics, Single‐Cell Center, Qingdao Institute of Bioenergy and Bioprocess Technology Chinese Academy of Sciences Qingdao China; ^2^ University of Chinese Academy of Sciences Beijing China; ^3^ Shandong Energy Institute Qingdao China; ^4^ Qingdao New Energy Shandong Laboratory Qingdao China; ^5^ Qingdao Single‐Cell Biotechnology Co. Ltd. Qingdao China

**Keywords:** artificial intelligence, One‐Cell‐One‐Tube (OCOT), single‐cell analysis, single‐cell printing, single‐cell sorting

## Abstract

Identification, sorting, and sequencing of individual cells directly from in situ samples have great potential for in‐depth analysis of the structure and function of microbiomes. In this work, based on an artificial intelligence (AI)‐assisted object detection model for cell phenotype screening and a cross‐interface contact method for single‐cell exporting, we developed an automatic and index‐based system called EasySort AUTO, where individual microbial cells are sorted and then packaged in a microdroplet and automatically exported in a precisely indexed, “One‐Cell‐One‐Tube” manner. The target cell is automatically identified based on an AI‐assisted object detection model and then mobilized via an optical tweezer for sorting. Then, a cross‐interface contact microfluidic printing method that we developed enables the automated transfer of cells from the chip to the tube, which leads to coupling with subsequent single‐cell culture or sequencing. The efficiency of the system for single‐cell printing is >93%. The throughput of the system for single‐cell printing is ~120 cells/h. Moreover, >80% of single cells of both yeast and *Escherichia coli* are culturable, suggesting the superior preservation of cell viability during sorting. Finally, AI‐assisted object detection supports automated sorting of target cells with high accuracy from mixed yeast samples, which was validated by downstream single‐cell proliferation assays. The automation, index maintenance, and vitality preservation of EasySort AUTO suggest its excellent application potential for single‐cell sorting.

## Impact statement

In this work, we developed an automatic and index‐based system called EasySort AUTO for single‐cell identification and sorting of microbial cells. Under a positive‐mount microscope, target single cells are identified via image‐based artificial intelligence algorithms and then moved from cell populations via optical tweezers, followed by exportation to PCR tubes through an automatic collection platform. During this process, a single cell is packaged in a microdroplet and automatically exported in a precisely indexed, “One‐Cell‐One‐Tube” manner. As it is automated, index‐based, and vitality preserved, the EasySort AUTO system can find broad applications in supplying target single cells from microbiome samples for single‐cell sequencing or cultivation.

## 1 INTRODUCTION

Single‐cell analysis has become a powerful tool for life sciences. Isolation of desired individual cells from a population or consortium is a key step in single‐cell analysis^1^, ^2^. This process can be generally achieved via fluorescence‐activated cell sorting (FACS) and shows great potential for in‐depth analysis of the structure and function of the microbiome when it links downstream single‐cell sequencing directly from an in situ sample^3^, ^4^, ^5^. However, it's usually complex in operation and cost demanding when we sort bacteria cells (10‐fold smaller in size than a typical human cell) using a FACS system at single‐cell precision. Moreover, a FACS system is generally unable to sort cells in an index‐based, “what you see is what you get” manner with highly vitality preserved. Although the dielectrophoresis (DEP)‐array strategy that couples the optical system with DEP can achieve reliable single‐cell identification and sorting^6^, it is extremely challenging for single‐cell sorting of microbial cells. Therefore, a simple, index‐based, and vitality‐preserved single‐cell sorting system is highly valuable for single‐cell analysis of microbiomes.

Microfluidics offers a promising solution for this problem^7^, ^8^, ^9^, ^10^, ^11^. Microfluidic systems can identify cells based on images^12^, ^13^, fluorescence signals^14^, ^15^, ^16^, or Raman spectrum^17^, ^18^, ^19^, and further sort the target cells via DEP^20^, ^21^, gravity^22^, centrifugal force^23^, optical tweezer^18^, ^24^, ^25^, or acoustic waves^26^, ^27^. However, sorted cells usually need to be flushed out of the chip for further omics analysis, which requires new tools to precisely dispense target cells to bridge single‐cell multiomics. An image, of either bright field, fluorescence, or Raman microspectroscopy, harbors rich phenotypic information to guide the cell sorting^12^. Image‐based cell sorting is a high‐resolution and label‐free detection method that allows single‐cell sorting directly from the original sample^28^, ^29^. We have developed a Raman‐activated gravity‐driven single‐cell encapsulation (RAGE) system^30^, where cells are screened via single‐cell Raman spectroscopy (SCRS) and then the target microbial single cells are obtained in a precisely indexed, “One‐Cell‐One‐Tube (OCOT)” manner that preserves their vitality (in the case of Raman‐based sorting). However, maintaining the aqueous phase static in the microchannel is a tedious operation, which is important for the high accuracy of single‐cell capture and encapsulation. To simplify the operation, recently, we developed an optical tweezer‐assisted pool‐screening and single‐cell isolation (OPSI) system for precise, indexed isolation of individual bacterial, yeast, or human cancer cells^31^. However, real‐time imaging, sorting, and exporting of cells still require tedious manual operations. To address these issues, two problems need to be solved: (i) cell detection and sorting still rely on manual selection and operation and (ii) target cells need to be manually exported from the chip.

Artificial intelligence (AI) is a promising tool for image analysis due to the capacity to detect inherent cellular properties hidden in cell images for cell identification^32^, ^33^. For example, cell species and stages in a malaria‐infected blood sample can be directly identified at single‐cell resolution based on bright‐field microscopy images^34^. Therefore, an AI‐assisted object detection model has the potential to realize cell identification and sorting automatically. Strategies such as inkjet‐like printing^35^, microfluidic impact printing^36^, and DEP array^6^ have been introduced in the automatic exporting of single cells; however, these strategies require complex chip fabrication or external control devices, which markedly increases the difficulty of instrument development and the cost of use. Therefore, an automated and low‐cost single‐cell export method still needs to be developed.

In this study, first, we used an AI‐assisted object detection model for automated cell identification. A one‐stage deep convolutional neural network (DCNN) was used for automated cell identification, which combines accuracy and speed. For automation of single‐cell exporting, we developed a cross‐interface contact method that does not require additional complex equipment and requires only a capillary tube connected to the chip outlet. Based on these two techniques, we developed an automatic and index‐based system called EasySort AUTO, by which cells of different morphology can be precisely sorted from cell populations and deposited as microdroplets containing a single cell in an OCOT manner. It is noteworthy that the system is built by simply coupling the optical tweezer module and the single‐cell collection module to a positive‐mount microscope platform that is already commonly found in biological laboratories. The throughput of the system for single‐cell printing is ~120 cells/h. The efficiency of the system for single‐cell printing is >93%. Subsequent single‐cell culture experiments showed that >80% of the isolated single cells of yeast or *Escherichia coli* could be recultured, suggesting the excellent preservation of cell viability during sorting. Finally, AI‐assisted image identification realized single‐cell sorting from mixed yeast samples, as confirmed by downstream single‐cell proliferation assays. As it is automated, simple, index‐based, and vitality‐preserved, the EasySort AUTO system showed great potential for broad applications, for example, by supplying target single cells from microbiomes for downstream sequencing or cultivation.

## 2 RESULTS

### 2.1 Design and working principle of the EasySort AUTO system

**Figure 1:**
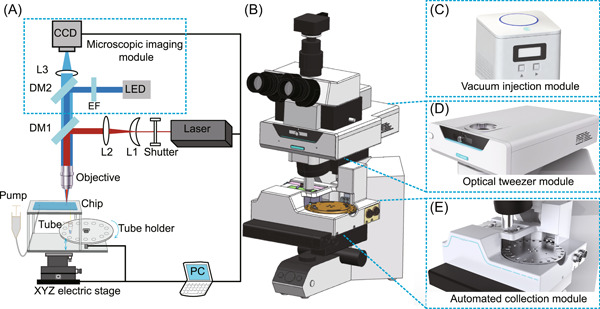
Overview of the EasySort AUTO system. (A) Schematic diagram of the optical path. (B) Schematic diagram of the instrument. (C) Diagram of the vacuum injection module. (D) Diagram of the optical tweezer module. (E) Diagram of the single‐cell collection module. CCD, charge‐coupled device; DM1, DM2, dichroic mirror; EF, excitation filter; L1, planoconcave lens; L2, planoconvex lens; L3, lens.

In this study, we developed a system called EasySort AUTO for automated single‐cell sorting (Figure 1A,B). The system consists of four main modules: a vacuum injection module for vacuum‐assisted sample loading (Figure 1C), a microscopic imaging module for image‐based observation of cells (Figure 1A), an optical tweezer module for manipulation of single cells (Figure 1D), and a single‐cell collection module for automated single‐cell printing (Figure 1E). The system was built by coupling the optical tweezer module and the automatic collection module to positive‐mount microscopy. Following the miniaturization and modularization principles, these modules can be readily installed in a wide range of microscope models from major commercial brands such as Olympus, Nikon, Leica, and Zeiss.

### 2.2 Design of the EasySort AUTO chip

**Figure 2:**
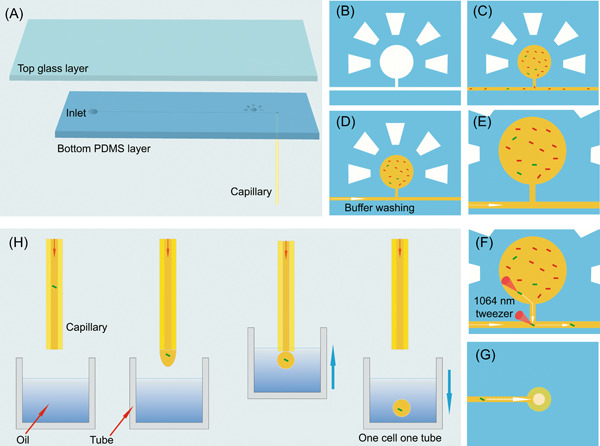
Scheme of the AI‐assisted automated single‐cell sorting and printing procedures in EasySort AUTO. (A) The chip was fabricated by bonding a top glass layer to a bottom PDMS layer coupled with a capillary. (B) Emptying the microchamber. (C) The microchamber was loaded with cell suspension after vacuuming treatment. (D) The microchannel was washed by the buffer after cell loading. (E) Automated image recognition of cells within microchambers. (F) The target single cell was moved to the microchannel by a 1064 nm optical tweezer. (G) The target single cell was driven by the fluid to flow towards the outlet. (H) The target single cell was encapsulated in the form of OCOT within the tube. AI, artificial intelligence; OCOT, One‐Cell‐One‐Tube; PDMS, polydimethylsiloxane.

The EasySort AUTO chip was designed following our previous OPSI chip, with some modifications. The chip is bonded by a top glass layer and a bottom polydimethylsiloxane (PDMS) layer that is coupled with a capillary (as the outlet) (Figure 2A). The bottom PDMS layer has a microchannel structure with a 1.5 mm diameter hole punched at one end of the channel as the inlet. A microchamber with a diameter of about 800 μm is connected to the main channel via a side channel (Figure 2B). The microchamber is designed for the storage and imaging of cells in the chip and is surrounded by multiple side chambers to assist in the filling of the microchamber with cell suspension (Figure 2C). Then, the buffer is injected into the main channel from the inlet to wash out the residual cells in the channel (Figure 2D). Cells in the microchamber are relatively static (Figure 2E).

For single‐cell sorting and printing, the identified target cells were captured individually by the optical tweezer and moved to the intersection of the main channel and the side channel by the software‐controlled platform (Figure 2F). Subsequently, the target single cell is driven by the fluid to flow toward the outlet (Figure 2G). After a certain duration, a droplet containing the target single cell is generated at the end of the capillary, and the operation of OCOT is achieved by moving upwords a tube containing oil to contact the capillary (Figure 2H). The contact between a capillary to the oil interface allows the transfer of a tiny amount of liquid from the capillary to the tube to realize single‐cell sorting.

### 2.3 Automated single‐cell sorting

**Figure 3:**
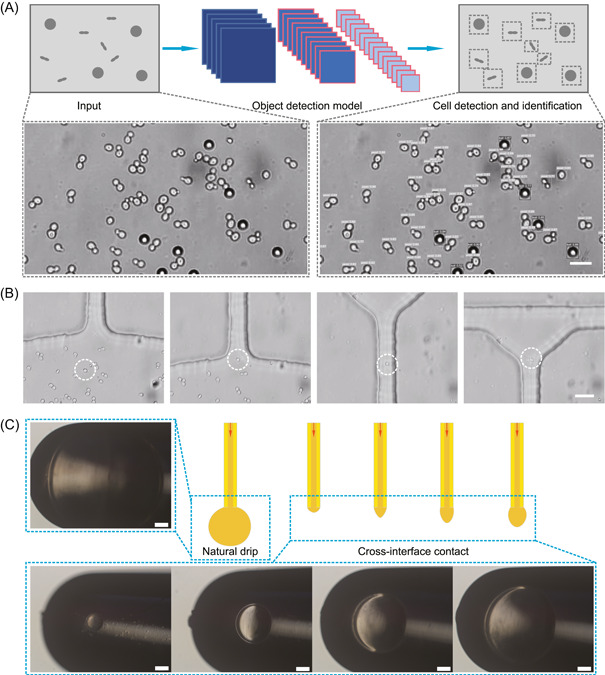
Automated single‐cell sorting in EasySort AUTO. (A) Cell detection and identification by the object detection model. Scale bar = 15 μm. (B) Automatic capture and movement of the targeted single cell by an optical tweezer. Scale bar = 20 μm. (C) Single‐cell printing by cross‐interface contact. Different volumes of liquid at the end of the capillary were simply transferred into the PCR tube by the cross‐interface contact method. Since the tube was previously prefilled with mineral oil, the liquid was encapsulated in the oil phase to form droplets. The images show the different‐size droplets in the PCR tube. Scale bar = 200 μm.

Step 1: Cell detection and identification by the object detection model. In this study, a mixed sample of yeast cells and microspheres is loaded into a microchamber in EasySort AUTO chip. An image of the bright field is captured by the camera and then transmitted to the computer (Figure 3A). Cell detection and identification are achieved by the object detection model. Yeast cells and microspheres in the field of view are accurately detected. The target cells can be automatically distinguished using the AI‐assisted object detection model (Video S1). In addition, this system provides a way to manually select the target cells of interest in the viewing field and then obtain the target cells by automated single‐cell printing.

Step 2: Automatic capture and movement of a targeted single cell by an optical tweezer. Before sorting, the system automatically identifies the start point (X1) and the endpoint (X2) of the movement path (Video S2), which is a fixed path for cells to move from the microchamber to the main channel. In addition, when a target cell is identified, the cell is rapidly located and the optical tweezer focus is then shifted quickly to the target‐cell location together with the turning on of laser to achieve cell capture. Subsequently, the optical tweezer drags the cell towards the start point (X1) of the well‐set path and moves along the fixed path to the endpoint (X2) (Figure 3B).

Step 3: Single‐cell printing by cross‐interface contact. The sorted target cells then flow to the end of the capillary driven by the main channel buffer fluid. The time required for the cells to travel from the microchamber to the end of the capillary is related to the flow rate of the buffer and the inner diameter of the capillary. Without the intervention of external conditions, the fluid at the end of the capillary accumulates to a volume of ~6 μl before dripping off under the force of gravity (Figure 3C). To achieve the on‐demand printing of cells, we developed a microfluidic printing method with cross‐interface contact. By contact of the capillary with the liquid surface, the interfacial energy, together with the fluid shear force generated by the liquid at the end of the capillary during the exchange of gas–liquid interface, overcomes the surface tension and adhesion of the liquid at the exit of the capillary end, so that the liquid at the end of the capillary can be separated from the capillary for liquid separation. The process is automated by software control and the volume of the droplets is flexible and adjustable (Figure 3C). The single‐cell collection module is activated by calculating the time of a single cell flowing to the end of the capillary to complete the printing of single cells.

### 2.4 Optimization of the system parameters

**Figure 4:**
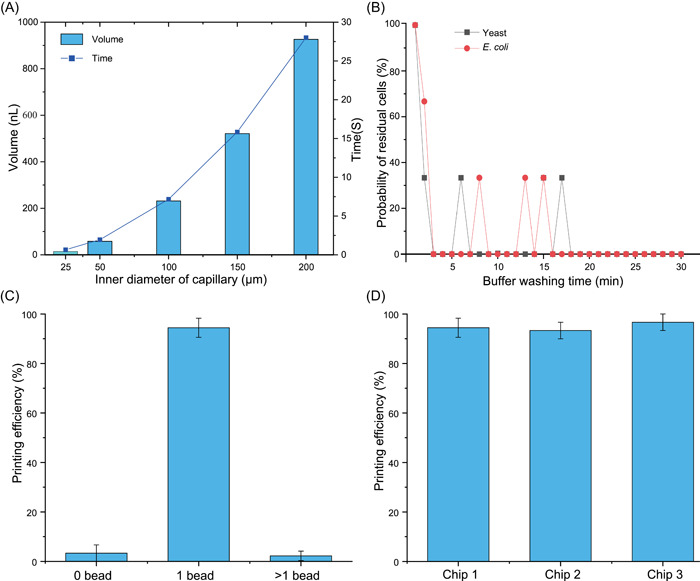
Optimization of the system parameters in EasySort AUTO. (A) Dead volume of the capillary with different inner diameters and the theoretical time required for cell flow through the capillary. (B) Time optimization for buffer washing after sample loading. (C) Printing efficiency in single‐cell sorting. (D) The efficiency of the system for single‐cell printing, as assessed by repeating the above operation with the three chips.

Cells can be sorted out of the microchamber in no more than 30 s based on the optical tweezer's force. The flow rate of the buffer was set to 2 µl/min to reduce the volume of exported droplets as much as possible. Limited by the distance between the microscope moving stage and the focusing device, the length of the capillary tube connected at the end of the chip is 29.5 mm, so as to ensure that the liquid at the end of the capillary tube was in contact with that inside the tube. For the selection of the capillary inner diameter, the volume at different inner diameters with the same length was determined (Figure 4A). The smaller the inner diameter of the capillary, the smaller the dead volume produced, and the shorter the time required for the chip‐to‐tube transfer of a cell. Meanwhile, in the case of use of a capillary injection, the efficiency of the injection is lower when the inner diameter of the capillary is too small. Consequently, the inner diameter of the capillary is set as 50 μm.

Sample loading in the chip is based on a self‐loading mechanism, which uses degas‐driven flow to draw the cell suspension through the capillary into the cell chamber. As a test, cells remaining in the channel were washed with buffer before subsequent single‐cell sorting experiments. After sample loading, the channels were washed using the buffer and the washed out liquid was collected every minute on the solid culture plate for incubation. Two types of commonly used microorganism samples, yeast and *E. coli*, were loaded into the chip separately for characterization. On counting the colony growth on the solid plates at various buffer washing time points (Figure 4B), it was found that few cells remained in the channel after 2 min of buffer washing. The probability of cell residue after 20 min of buffer washing was zero, indicating that the residual cells in the channel had been completely washed out within the duration.

### 2.5 Evaluation of the accuracy and reliability of the EasySort AUTO system

For single‐cell analysis, it is not desirable to include two or more cells in a droplet. Therefore, the ability to accurately and reliably separate single cells is key for single‐cell analysis systems. We tested the ability to dispense single cells of this optimized system using fluorescent microspheres. The fluorescent microsphere was removed from the microchamber by the optical tweezer, flown to the end of the capillary by the buffer, and transferred to a 0.2 ml PCR tube using an automatic droplet collection device. The printing efficiency was calculated by fluorescence imaging under a microscope. The printing efficiency of droplets is defined as the ratio of the number of droplets containing the corresponding number of beads to the total number of droplets. Experiments were conducted using fluorescent microspheres with a diameter of 5 µm. Single‐cell sorting was performed using this system, and a total of 90 fluorescent microspheres were removed in three groups and collected separately into test tubes to count the printing efficiency. The printing efficiency of the droplets containing a single bead was ~94% (Figure 4C). The throughput of the system for single‐cell printing is ~120 cells/h. On repeating the above operation with three separate chips, it was found that the efficiency of the system for single‐cell printing is above 93% (Figure 4D). Therefore, the system can print single microbial cells stably and reliably.

### 2.6 Single‐cell sorting in an OCOT manner

**Figure 5:**
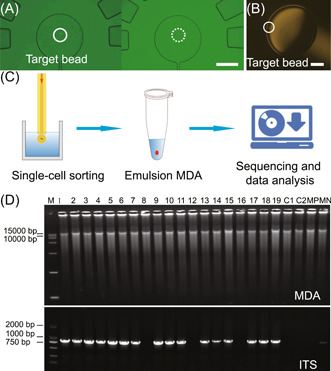
Single‐cell printing for single‐cell genomic analysis. (A) The target fluorescent microspheres can be identified rapidly by microscopic fluorescence imaging, and the percentage of target cells was approximately one‐thousandth of the total. Scale bar = 400 μm. (B) The target fluorescent microsphere was collected in the tube. Scale bar = 300 μm. (C) Workflow that combines the EasySort AUTO platform with single‐cell sequencing. (D) One‐cell MDA reactions from 19 EasySort AUTO‐sorted yeast cells. Two empty droplets (C1 and C2 as negative controls) were also amplified. 1–19, the one‐cell droplet samples; M, DNA ladder. Samples 7, 12, and 16 failed to show positive amplicon in ITS. ITS, internal transcribed spacer; MDA, multiple displacement amplification. MP, MDA Positive; MN, MDA Negative.

The system was coupled with a 1064 nm optical tweezer for precise automated single‐cell sorting of cells from 1 to 30 μm in diameter. To demonstrate the capability of the system for single‐cell sorting, fluorescent microspheres of 5 μm diameter as target cells were mixed with pigmented yeast and loaded into microchambers. As shown in Figure 5A, the target fluorescent microsphere account for approximately one‐thousandth of the total was rapidly identified by microscopic fluorescence imaging. Fluorescence‐based imaging combined with automated sorting with an optical tweezer was used to collect the target microsphere in the tube (Figure 5B). The sorted cells in the form of OCOT can be directly used for subsequent single‐cell genome analysis.

To demonstrate the system's ability to support single‐cell genome analysis, a single‐cell sorting‐sequencing workflow was developed based on EasySort AUTO by combining the platform with single‐cell DNA amplification (Figure 5C). The encapsulation and isolation of single cells in a small volume would improve downstream single‐cell sequencing, for example, by reducing the bias of amplification and increasing the genome‐wide sequence coverage, as demonstrated in our previous study^30^. Thus, to verify the system workflow, only multiple displacement amplification (MDA) reactions were performed. After the addition of lysis reagents to the PCR tubes containing single cells, a transient centrifugation is used to release cells from the original droplets, resulting in good mixing with lysis reagents and stop solution successively, to complete single‐cell lysis and nucleic acid denaturation. Then, the MDA reagent is added to the tube via transient centrifugation to evoke single‐cell genome amplification for internal transcribed spacer (ITS) sequencing. In total, we tested 19 yeast cells, among which 16 produced ITS‐positive bands and 3 produced ITS‐negative bands (Figure 5D). Problems in cell lysis might be a reason why ITS‐negative bands were observed. ITS sequencing results confirmed that all the ITS‐positive products were from yeast, implying that the success rate of single‐cell sorting‐sequencing via our system was at least 84.2%.

**Figure 6:**
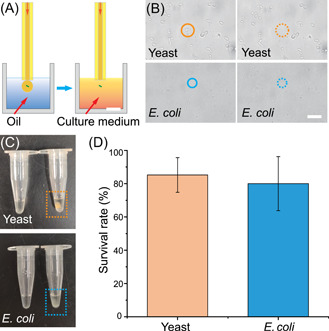
Single‐cell printing coupled with the single‐cell proliferation assay. (A) For single‐cell proliferation, the mineral oil in the tube was switched to the culture medium for growth. (B) Yeast cells and *Escherichia coli* within the microchamber were identified and picked out from the microchamber by the optical tweezer. Scale bar = 20 μm. (C) After 48 h of incubation, the liquid in the PCR tube appeared muddy, indicating that cell proliferation had occurred. (D) The survival rate of single‐cell proliferation was approximately 85% for yeast cells and 80% for *E. coli* cells.

For many applications, the successful proliferation of isolated single cells is important. To ensure the viability of sorted cell, the mineral oil in the tube is replaced by a medium suitable for cell growth and proliferation (Figure 6A). When performing the cultivation, the target single cell was directly transferred to the PCR tube without droplet formation. This allows a single cell to be released into the medium without going through the oil phase encapsulation, thus avoiding the influence of mineral oil on cell cultivation. As a test, yeast or *E. coli* cells within the microchamber were identified, picked out from the microchamber by an optical tweezer, and then driven by the flowing buffer to the end of the capillary and collected into PCR tubes containing 20 µl of medium (Figure 6B). After collection, the PCR tubes containing single cells were incubated in a constant temperature shaker. Three groups, each including 20 individual cells (OCOT), were collected separately and cultured in PCR tubes. After 48 h of incubation, the liquid in the PCR tube appeared muddy, indicating successful single‐cell proliferation (Figure 6C). As shown in Figure 6D, the success rate of single‐cell proliferation (survival rate) after EasySort AUTO was ~85% for yeast cells and 80% for *E. coli* cells.

### 2.7 AI‐assisted single‐cell sorting and proliferation

**Figure 7:**
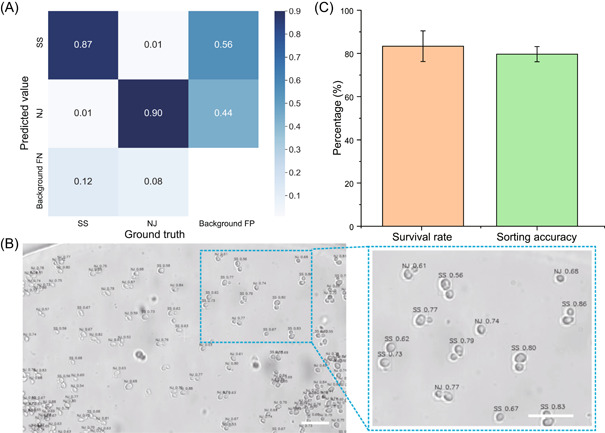
AI‐assisted single‐cell sorting and proliferation. (A) Confusion matrix of the model in recognizing the cells. Background FP is misidentifying the background as the target and Background FN is misidentifying the target as the background. (B) Automatic identification of the target yeast cells. Scale bar = 30 μm. (C) Survival rate calculated by single‐cell culture and the sorting accuracy calculated based on single‐cell genome sequences. AI, artificial intelligence; FN, false negative; FP, false positive; NJ, noncarotenoid‐producing *Saccharomyces cerevisiae* BY4742; SS, carotenoid‐producing *Phaffia rhodozyma* ATCC 24202.

An AI‐assisted object detection model can quickly acquire morphological images of individual cells and present predicted results. In this study, we used two kinds of yeasts that differ morphologically to validate the accuracy of identification. First, we collected photographs of two kinds of yeast cells under the microscope, including carotenoid‐producing *Phaffia rhodozyma* ATCC 24202 (designated as SS) and noncarotenoid‐producing *Saccharomyces cerevisiae* BY4742 (designated as NJ; Figure S1). These two kinds of yeasts are different in brightness, shape, and texture. SS has less contrast, a rounded shape, and no significant texture variation internally as compared to NJ. These characteristics can be used as recognition features extracted by the AI‐assisted object detection model. Then, the object detection model was modified according to the actual data set and trained using the pretrained weights file. The vast majority of SS and NJ were classified correctly (Figure 7A).

Then, the carotenoid‐producing yeast cells and noncarotenoid‐producing yeast cells were mixed in a ratio of 1:10 and loaded into the chip for single‐cell imaging and sorting (Figure 7B). Precisely 23 carotenoid‐producing yeast cells were recognized by the object detection model and then exported in an OCOT manner into a growth medium. The operation was repeated three times, resulting in 69 carotenoid‐producing cells. After incubation, the precipitate at the bottom of the tube that appears orange is SS (highlighted by an orange dotted line), while the precipitate that appears white is NJ (highlighted by a white dotted line) (Figure S2). The average survival rate of the yeast single cells was ~83%, confirming the superior preservation of cell vitality in EasySort AUTO operation. Comparison between the image‐recognition‐derived prediction and validation based on postculture suggests that the overall accuracy of image‐recognized‐based discrimination between the two types of yeast cells is ~80% (Figure 7C).

## 3 DISCUSSION

In this work, using an AI‐assisted object detection model for cell phenotype screening and a cross‐interface contact method for single‐cell exporting, we developed an automatic and index‐based system called EasySort AUTO, where individual microbial cells are sorted, then packaged in microdroplets and automatically exported in a precisely indexed “OCOT” manner. In this system, the microfluidic chip is designed with a microchamber for cell detection and identification and a capillary for automated single‐cell exporting. The AI‐assisted object detection model achieved highly accurate recognition of target cells from mixed samples loaded in the microchamber. The system‐integrated optical tweezer module permits precise movement of cells with sizes ranging from 1 to 30 μm at a damage‐free and high manipulation resolution. The single‐cell collection module enables the automated transfer of cells from the chip into the tube, thus readily coupled with subsequent single‐cell culture or sequencing.

The major difference of AI algorithms compared to traditional image recognition algorithms is that it automatically extracts features using convolutional kernel and backpropagation. The AI‐assisted single‐cell sorting system enables the automatic identification of target cells from mixed samples. AI‐assisted cell identification replaces manual selection to achieve automated cell identification. The integrated 1064 nm optical tweezer achieves cell sorting in a wide size range of 1–30 µm. In addition, the strategy of performing static cell imaging in a microchamber, which does not limit the imaging interrogation time, is particularly important and desirable for precise yet massively paralleled screening of those rare target cells from a highly heterogeneous sample. Furthermore, the cross‐interface contact method enables the automated transfer of cells within the chip into a macroscopic tube. We showed that >80% single cells of both yeast and *E. coli* are culturable after such sorting, indicating superior preservation of cell viability in sorting. Finally, AI‐assisted object detection supports automated, high‐accuracy sorting of target cells from mixed yeast samples, as confirmed by downstream single‐cell proliferation assays.

FACS can be used for microorganism identification based on image, fluorescence, scattering light, and in very high throughput. However, FACS requires expensive equipment, professional manual operation, and tedious sample preparation to ensure the accuracy of experimental results, which is challenging for the general experimenter to perform the single‐cell sorting. In comparison, the EasySort AUTO system's modular design and versatility in coupling with light field, fluorescence, or Raman spectroscopes will greatly improve the accessibility of microbial single‐cell omics to small laboratories. Nonetheless, the viability of the FACS‐sorted cells can be negatively affected by numerous factors of instruments, such as mechanical and physiological stresses during sorting, making recovery of the desired cells difficult. In contrast, the EasySort AUTO sorted cells maintained superior preservation of cell viability. Notably, when the system performs automated image recognition of small‐sized bacteria, a proportion of the bacterial cells may not be detected if the cell is not close to the focal plane. This can be resolved by designing smaller chamber‐depth chips to improve the visual clarity of very small microbial cells or by integrating additional mechanisms for focusing. In addition, when the system performs the automated movement of the target cell, nontarget cells on the route can potentially interfere with the target‐cell movement. To tackle this problem, we need to implement an algorithm for path planning that considers such possibilities.

The throughput of the EasySort AUTO system for single‐cell printing is ~120 cells/h at present. The low speed of cell manipulation using optical tweezers and the low efficiency of automated single‐cell export are two major limiting factors. For the former, it is possible to increase the movement speed of the platform and the laser intensity to ensure that the optical tweezers can consistently and rapidly manipulate the cells. For the latter, although the cross‐interface contact method requires very simple equipment to achieve single‐cell droplet export, the low movement speed of the system's automated collection module limits the speed of single‐cell collection. To increase the throughput, a new three‐dimensional moving platform with higher collection speed and accuracy is in development for automated single‐cell collection.

The EasySort AUTO system represents an automated, index‐based, and vitality‐preserved solution for microbial single‐cell sorting. Although we have demonstrated the adaptability of image‐based microbial screening through a mixture of two strains, the application of image‐based microorganism screening is still limited because the same bacterial strain can have different sizes and shapes, and different microorganisms possibly have very similar sizes and shapes. When using AI‐assisted image recognition algorithms for image recognition, both sample origin and imaging quality can affect the accuracy of recognition. Stable and significant visual differences are also essentially required thus AI algorithm could extract for identification. The wide application of image‐based microbial screening requires the development of clearer microbial image cameras and smarter AI‐assisted image recognition algorithms, which is still challenging. In addition, the fluorescence‐based sorting provided by the system can be combined with fluorescent labeling methods such as fluorescence in situ hybridization (FISH) for downstream genomic analysis to obtain microbial taxonomic and functional information. Notably, the system has the potential to be coupled with a Raman spectroscope for single‐cell sorting, which will considerably broaden the application of the system. The ability of the system to link phenotypes with genotypes provides a good tool for further single‐cell analysis.

## 4 MATERIALS AND METHODS

### 4.1 System setup of the EasySort AUTO

The EasySort AUTO instrument (Qingdao Single‐cell Biotechnology Co., Ltd.) was designed for automatic single‐cell imaging, manipulation, and sorting (schematic diagram of the optical path in Figure 1A). A 1064 nm continuous‐wave diode‐pumped laser (Changchun New Industries Optoelectronics Technology Co., Ltd.) was used as the light source of the optical tweezer for single‐cell manipulation. The laser beam was expanded with a beam expander (3 ×) that consists of L1 and L2. A 50 × objective lens (NA 0.7; Olympus) was used to focus the laser beam via a dichroic mirror (DM1) into the chip perpendicularly to trap the cell. A light‐emitting diode (LED) was used as the fluorescence light source, and then the LED light traveled through a filter set (excitation filter EF and dichroic mirror DM2) into the objective lens to illuminate the sample. In this study, two filter sets were used to capture the yellow and red fluorescence images of the sample via a charge‐coupled device (CCD) camera. The automated collection module consisted of three parts: a tube holder, an electric motor, and a three‐axis motorized translation stage. The tube holder includes 24 holes to install the tubes, and it can rotate with an electric motor to collect single cells accurately. The images were processed in real time using a computer to identify target objects, for example, a single cell, and then a trigger signal was sent to the signal control module, which triggered the optical tweezer to trap the cell.

### 4.2 Chip design and fabrication

The chip structure was designed using AutoCAD 2019 (Autodesk Inc.). The PDMS layer was fabricated using soft lithography and rapid prototyping techniques. Specifically, a ~40‐μm‐thick microstructure mold was fabricated on a silicon wafer. A PDMS prepolymer with curing agent mixed in a 10:1 ratio (SYLGARD 184; Dow Corning) was poured onto a mold prepared and baked at 70°C for 2 h to ensure complete curing. The PDMS was peeled off from the mold, and a through‐hole 1.5 mm in diameter was punched at the main‐channel tip for the inlet tube connecting. A small hole was punched at the other end using a 400 µm hole punch as the outlet. Due to the flexible deformation of PDMS during punching, the internal diameter of the through‐hole punched by the puncher would be slightly less than 400 µm. The PDMS layer was bonded with a 1‐mm‐thick glass slide by oxygen plasma. After fabrication, the chip was placed overnight in a 70°C oven to strengthen bonding. A length of 29.5 mm quartz capillary was cut and inserted into the chip outlet so that the liquid at the outlet could flow from the end of the capillary.

### 4.3 Cell culture and sample preparation

All reagents were purchased from Sigma‐Aldrich, except when specified otherwise. Green fluorescent protein (GFP) expressing *E. coli* (DH5α) were cultured on Columbia blood agar plates at 37°C overnight. Budding yeast strains *S. cerevisiae* BY4742 (designated as NJ) and *P. rhodozyma* ATCC 24202 (designated as SS) were cultured on yeast extract‐peptone‐dextrose (YPD) agar plates at 30°C for 24 h. A single colony was inoculated into PBS solution and diluted to a concentration of ~10^7^ cell/ml. Pluronic F127 (1% wt) was added to suspensions to avoid cell adhesion in the chip.

The chip inlet was sealed with adhesive tape, and then the chip was placed in the vacuum injection module for 10 min of vacuum treatment. After the chip was removed from the vacuum chamber, the end of the chip capillary was immediately placed in a PCR tube containing a cell suspension (10–50 μl). After the microchamber was filled with the sample, the buffer was connected to the chip inlet to wash the residual cells in the channel out of the chip. Finally, the loaded chip was placed on the microscopic stage of the EasySort AUTO platform for observation. For single‐cell omics analysis, mineral oil with 2% wt EM90 was added to the PCR tube from the collection module for generating droplets containing single cells. For single‐cell culture, an appropriate amount of medium solution was added to the PCR tube that harbors the target cell for subsequent single‐cell culture.

### 4.4 AI‐assisted cell detection and identification

Image‐based cell sorting is a high‐resolution and label‐free detection method that allows single‐cell sorting directly from the original sample. AI‐assisted image recognition has three main steps: first, feature extraction by a convolutional operation, then feature synthesis and judgment by the neural network, and finally, use of a backpropagation algorithm to modify convolutional kernel and neural network parameters to optimize the judgment. Specifically, DCNN was used for automated cell identification. The model used was YOLOv5, which combines accuracy and speed, and originated from YOLOv1^37^. First, we collected photographs under the microscope. Then, the model configuration was modified according to the actual data set and trained using a pretrained weights file. After capture, the image was first adjusted, including padding and format conversion, to match the input expectations of the model. The training set contains two sets of photos: one containing 105 samples and the other containing 81 samples. The validation set also includes two sets of photos, respectively, containing 86 and 71 samples. A data set of this size would be too small to build a model from zero, so we use a pretrained model, which itself has been trained by the COCO data set. The image was then inferred using the neural network to obtain the detection target and the corresponding confidence level. The detection result at this point still contains many overlapping detection frames; thus, it is necessary to perform nonmaximum suppression deduplication and then draw the detection frames, as well as the corresponding categories and confidence levels, on the original image. Finally, the detected cell image was displayed on the graphical user interface (GUI). The time required for the recognition of the single cell is about 120 ms.

### 4.5 Single‐cell sorting

The system provides an automated sorting workflow to support downstream single‐cell analysis. In brief, the sample was loaded into the chip through a vacuum feed module that creates a negative pressure inside the chip. Then, the image of each cell inside the chip was acquired via microscopic imaging, and single‐cell identification was achieved by parallel machine‐learning algorithms. Actually, this system provides a flexible way to identify cells. For example, a parameter can be set, and the target cells can be identified automatically via an AI algorithm, along with the assignment of a number, as shown in Video S1. Meanwhile, this system allows the manual selection of interested cells in the view, such as cells with fluorescence. Subsequently, the target cells were captured and moved to the specific position by the optical tweezer module. Finally, the target cells were exported in an OCOT manner via the automatic collection module. Targeted single cells in the microscopic field of view can be collected into PCR tubes for a “What you see is what you get” operation. The photo of each cell was stored, and the sorted cells were numbered, which can be matched with the number of the collected tubes. The single cells in each tube are indexable.

### 4.6 Single‐cell genome sequencing and data analysis

Before the experiments, the surfaces of all equipment (objective platform, pump, solenoid valve, etc.) were cleaned with DNA AWAY™ (Molecular BioProducts). For DNA amplification, precisely one OCOT‐sorted yeast cell was centrifuged to the bottom of the PCR tube and the lysis reagents were added for cell lysis by gentle centrifugation. Neutralization solution was added by gentle centrifugation to neutralize the lysis mixture. Then, 15 µl of reagent mixture consisting of Reaction Buffer and phi 29 DNA polymerase from the SupreAmp scWGA Kit (Qingdao Single‐Cell Biotechnology Co., Ltd.) was added to the PCR tube, followed by incubation at 30°C for 8 h at a 70°C hot‐lid temperature for MDA reactions. A negative control (empty droplet without any cells) was also included to detect and quantify possible contamination.

For ITS sequencing, the MDA products were diluted in a ratio of 1:20–1:40. ITS primers (ITS4 and ITS5) that target the eukaryotic ITS gene were chosen for this study. The 25 µl PCR reaction mixture, which contains 12.5 µl of 2 × MIX, 1 µl of 10 µM ITS4 primer, 1 µl of 10 µM ITS5 primer, 9.5 µl of nuclease‐free water, and 1 µl of diluted MDA product as a template, was mixed by vortexing and spinning down. The PCR reaction program is 94°C for 30 s, 33 cycles of 98°C for 10 s, 55°C for 30 s, 72°C for 1 min, and 72°C for 5 min. The positive ITS products were sent for Sanger sequencing (Sangon Biotech). The ITS sequences were analyzed by the blast at NCBI (https://www.ncbi.nlm.nih.gov/).

## AUTHOR CONTRIBUTIONS

Zhidian Diao, Bo Ma, and Yuandong Li conceived the system. Zhidian Diao and Teng Xu designed the chip. Yuandong Li, Lingyan Kan, Huaibo Yang, and Yuetong Ji developed the EasySort AUTO instrument. Yilong Zhao coded the control software. Zhidian Diao, Jingyun Song, Chen Wang, Fengli Zhang and Rongze Chen performed the experiment. Zhidian Diao, Lingyan Kan, and Yilong Zhao wrote the paper. Yang Liu, Xiaoyan Jing, Xixian Wang, Bo Ma, and Jian Xu polished the paper. All the authors have given approval to the final version of the manuscript.

## ETHICS STATEMENT

No animal experiments were conducted in this study. There is no ethical approval to be declared.

## CONFLICT OF INTERESTS

Professors Jian Xu and Bo Ma are among the founders of Qingdao Single‐Cell Biotechnology Co., Ltd.

## Supporting information

Supporting information.

Supporting information.

Supporting information.

## Data Availability

The details of the system component and the control program in this work are available within the paper and its Supporting Information files.
