# Supplementary material for: Artificial intelligence‐assisted automatic and index‐based microbial single‐cell sorting system for One‐Cell‐One‐Tube
Source: mLife. 2022 Dec 18;1(4):448–59. doi: 10.1002/mlf2.12047 (PMC10989846; doi:10.1002/mlf2.12047)
Supplement: Supplementary file 1 — Supporting information. [file MLF2-1-448-s003.doc]

# Supplementary Information

**Artificial intelligence-assisted automatic and index-based microbial single-cell sorting system for One-Cell-One-Tube**

Zhidian Diao^1,2, #^, Lingyan Kan^1,2, #^, Yilong Zhao^1,2, #^, Huaibo Yang^3^, Jingyun Song^1,2^, Chen Wang^1,2^, Yang Liu^1,2^, Fengli Zhang^1,2^, Teng Xu^1,2^, Rongze Chen^1,2^, Yuetong Ji^1,3^, Xixian Wang^1,2^, Xiaoyan Jing^1,2^, Jian Xu^1,2^, Yuandong Li^1,2^, *, Bo Ma^1,2^, *

^1^ Single-Cell Center, CAS Key Laboratory of Biofuels, Shandong Key Laboratory of Energy Genetics, Shandong Energy Institute, Qingdao Institute of Bioenergy and Bioprocess Technology, Chinese Academy of Sciences, Qingdao, Shandong 266101, China.

^2^ University of Chinese Academy of Sciences, Beijing 100049, China.

^3^ Qingdao Single-Cell Biotechnology, Co., Ltd., Qingdao, China.

* Corresponding author

^#^ These authors contributed equally to this work.

Add: Single-Cell Center, CAS Key Laboratory of Biofuels, Shandong Key Laboratory of Energy Genetics, Shandong Energy Institute, Qingdao Institute of Bioenergy and Bioprocess Technology, Chinese Academy of Sciences, Qingdao, Shandong 266101, China

Email: mabo@qibebt.ac.cn (Bo Ma); Phone: +86-0532-80662657.

Email: liyd@qibebt.ac.cn (Yuandong Li); Phone: +86-0532-80662717.

Video S1: the process of identifying and sorting single cells.

Video S2: the cell movement route is automatically determined.


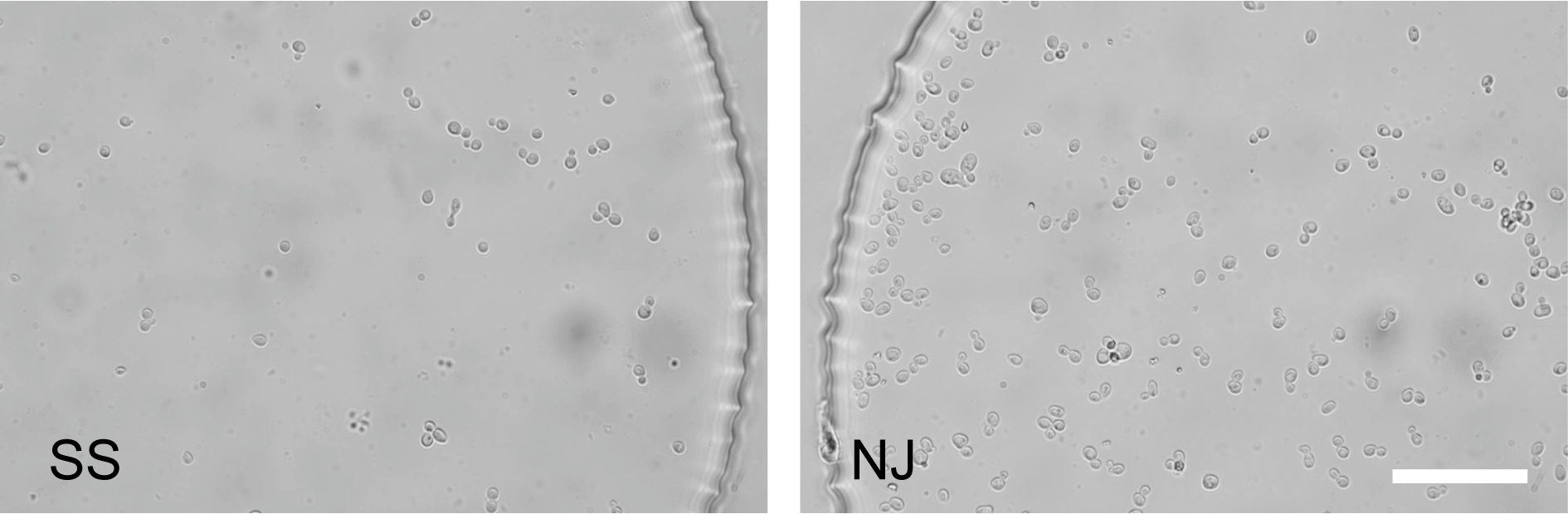


**Figure S1.** Photographs of two kinds of yeasts cells under the microscope, including the carotenoid producing *P. rhodozyma* ATCC 24202 (Marked as SS) and non-carotenoid producing *S. cerevisiae* BY4742 (Marked as NJ). Scale bar: 40 μm.


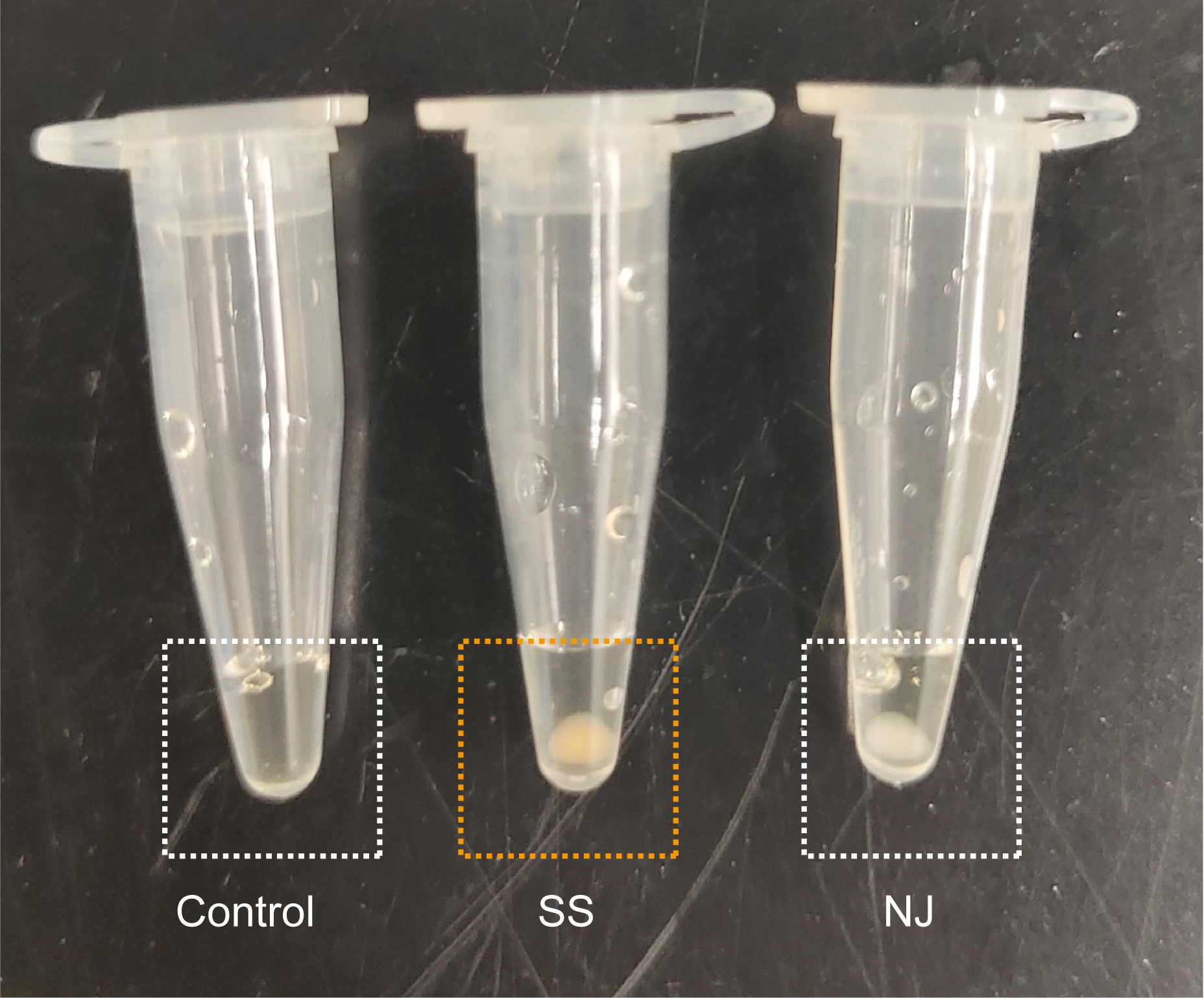


**Figure S2.** Precipitates collected after culture of sorted yeast cells. SS was highlighted by orange dotted line, and NJ was highlighted by white dotted line.
